# Alterations in Skeletal Muscle Cell Homeostasis in a Mouse Model of Cigarette Smoke Exposure

**DOI:** 10.1371/journal.pone.0066433

**Published:** 2013-06-14

**Authors:** Marc-André Caron, Mathieu C. Morissette, Marie-Eve Thériault, Jake K. Nikota, Martin R. Stämpfli, Richard Debigaré

**Affiliations:** 1 Centre de Recherche, Institut Universitaire de Cardiologie et de Pneumologie de Québec, and Laval University, Quebec City, Quebec, Canada; 2 Department of Pathology and Molecular Medicine, McMaster Immunology Research Centre, McMaster University, Hamilton, Ontario, Canada; 3 Firestone Institute for Respiratory Health at St. Joseph’s Healthcare, McMaster University, Hamilton, Ontario, Canada; University of Rochester Medical Center, United States of America

## Abstract

**Background:**

Skeletal muscle dysfunction is common in chronic obstructive pulmonary disease (COPD), a disease mainly caused by chronic cigarette use. An important proportion of patients with COPD have decreased muscle mass, suggesting that chronic cigarette smoke exposure may interfere with skeletal muscle cellular equilibrium. Therefore, the main objective of this study was to investigate the kinetic of the effects that cigarette smoke exposure has on skeletal muscle cell signaling involved in protein homeostasis and to assess the reversibility of these effects.

**Methods:**

A mouse model of cigarette smoke exposure was used to assess skeletal muscle changes. BALB/c mice were exposed to cigarette smoke or room air for 8 weeks, 24 weeks or 24 weeks followed by 60 days of cessation. The gastrocnemius and soleus muscles were collected and the activation state of key mediators involved in protein synthesis and degradation was assessed.

**Results:**

Gastrocnemius and soleus were smaller in mice exposed to cigarette smoke for 8 and 24 weeks compared to room air exposed animals. Pro-degradation proteins were induced at the mRNA level after 8 and 24 weeks. Twenty-four weeks of cigarette smoke exposure induced pro-degradation proteins and reduced Akt phosphorylation and glycogen synthase kinase-3β quantity. A 60-day smoking cessation period reversed the cell signaling alterations induced by cigarette smoke exposure.

**Conclusions:**

Repeated cigarette smoke exposure induces reversible muscle signaling alterations that are dependent on the duration of the cigarette smoke exposure. These results highlights a beneficial aspect associated with smoking cessation.

## Introduction

Chronic obstructive pulmonary disease (COPD) is a leading cause of disability [1] and currently ranks as the fifth cause of mortality worldwide [2]. COPD is associated with profound systemic repercussions, including skeletal muscle dysfunction [3]. Given its negative impact on quality of life [4], exercise tolerance [5], and survival prognostic [6], COPD-associated muscle dysfunction is of particular interest. While smoking remains the main causal agent of COPD in industrialized countries [1], little is elucidated on the impact of chronic cigarette use on skeletal muscle prior to lung disease development.

Inflammation and oxidized proteins are two suspected contributors to the development of skeletal muscle dysfunction observed in COPD. In humans, non-COPD smokers exhibit reduced type I fiber cross-sectional area, increased glycolytic enzymatic activity, as well as lowered endothelial and neuronal nitrite oxide synthase activities in their *vastus lateralis* when compared to non-smoking controls [7]. Current smokers present increased serum tumor necrosis factor alpha (TNF-α) [8], a potent inducer of skeletal muscle protein degradation [9]. These data suggest that cigarette use could contribute to the establishment of skeletal muscle dysfunction prior to pulmonary disease development. These findings are also reported in animals. Mice chronically exposed to cigarette smoke tend to show decreased muscle oxidative activity, along with a slight change in fiber type proportion [10], [11], [12]. Elevated circulating TNF-α levels are also reported in these models [10], [13]. Furthermore, reduced muscle capillary to fiber ratio has been observed in smoke-exposed mice [14], along with decreased vascular endothelial growth factor (VEGF) at the mRNA [13] and the protein levels [14]. Mice and guinea pigs exposed to cigarette smoke also exhibit increased levels of oxidized proteins in the gastrocnemius [15], [16].

**Figure 1:**
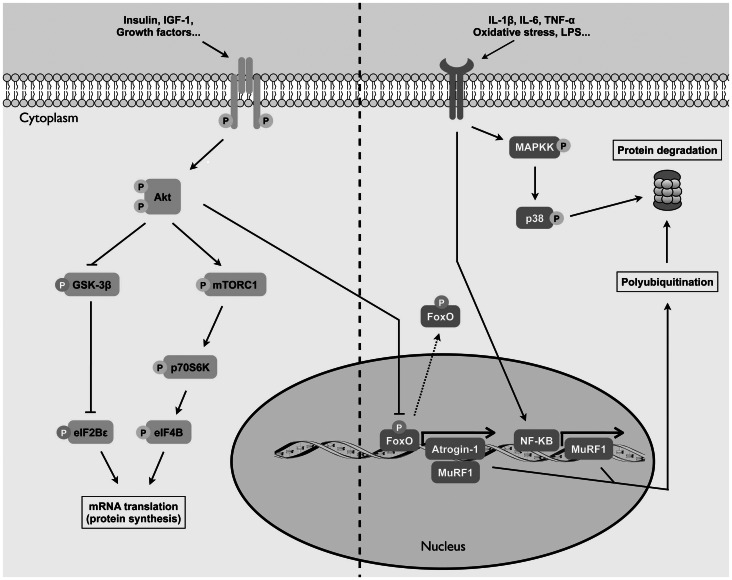
Akt and ubiquitin-proteasome signaling pathways. On the left side, upon stimulation of their respective receptor, insulin or insulin-growth factor 1 stimulates phosphorylation of Akt. Protein synthesis is then promoted through activation of p70S6K and inhibition of GSK-3β. On the right side, following stimulation by the appropriate stimuli (pro-inflammatory cytokines, oxidative stress, lipopolysaccharide, etc) NF-κB becomes activated and transcribes, among others, MuRF1. In pro-atrophic conditions, FoxO also transcribes MuRF1 and Atrogin-1. These E3-ligases increase total polyubiquitination and therefore promote protein degradation through the proteasome. Mitogen activated protein kinase p38 is also known to induce muscle protein degradation. In addition to promoting synthesis, activated Akt is an inhibitor of protein degradation by restraining nuclear translocation of FoxO. Activation = Filled arrows Inhibition = Hammer head lines Translocation = Dashed arrows.

Numerous cell signaling pathways have been highlighted over the years for their role in the development of skeletal muscle atrophy, a key element of muscle dysfunction [17]. Atrophy occurs when protein degradation exceeds protein synthesis. Wasting of the muscle tissue is associated with increased proteasomal activity [18], [19], along with higher levels of muscle-specific E3 ligases Atrogin-1 and Muscle RING finger 1 (MuRF1) [20], [21], [22]. The phosphatidylinisitol-3 kinase (PI3K)/Akt pathway also appears to play a significant role in skeletal muscle atrophy development as Akt exhibits a reduced activity in atrophying muscles [23]. Consequences of decreased Akt activity are, among others, a preserved inhibitory activity of glycogen synthase kinase-3 beta (GSK-3β) along with a decline in the activity of mammalian target of rapamycin complex 1 (mTORC1) and its downstream target p70 S6 kinase (p70S6K); both situations contribute to the impeded initiation of mRNA translation [24], [25], [26]. These pathways are summarized in Figure 1.

The main objective of this study was to further investigate the effects of cigarette smoke exposures on skeletal muscle cell signaling involved in protein synthesis and breakdown and to assess the reversibility of such effects. To achieve this aim, mice were exposed to cigarette smoke using a whole body cigarette smoke exposure system. The chosen cigarette exposure model is known to exhibit ventilation/perfusion mismatch after 8 weeks and to progress with significant neutrophilia and slight airspace enlargement after 24 weeks, which are hallmarks of COPD [27]. Two specific approaches were implemented to 1) investigate whether an atrophy-favorable cell environment is promoted through sub-chronic (8 weeks) and chronic (24 weeks) smoke exposure, and 2) assess the reversibility of the cellular response following smoking cessation (60 days). We hypothesized that cigarette smoke exposures would affect muscle cell signaling in favor of a pro-atrophic phenotype, and that these alterations would amplify when smoke exposures are prolonged from 8 to 24 weeks. Furthermore, smoking cessation would attenuate the smoke-induced cell signaling alterations. Part of this work has been published in the form of an abstract [28].

## Materials and Methods

### Animals

58 pathogen-free 6–8 week-old female BALB/c mice were purchased from Charles River Laboratories (Montreal, Qc, Canada). All animals were kept under a 12∶12-h light-dark cycle, with food and water provided *ad libitum*. The McMaster University Animal Research Ethics Board approved all the experiments performed on living animal and all efforts were applied to minimize discomfort.

### Study Design and Cigarette Smoke Exposures

In a first set of experiments, mice (9–10 per group) were exposed to cigarette smoke for 8 or 24 weeks as previously described in detail [29]. Mice were exposed to 12 2R4F reference cigarettes (Tobacco and Health Research Institute, University of Kentucky, Lexington, KY, USA) with filters removed, for a period of approximately 50 minutes, twice daily, 5 days/week, using a whole body smoke exposure system (SIU-48, Promech Lab AB, Vintrie, Sweden). Total particular matter (TPM) concentration in the exposure box was approximately 600 µg/l. We previously reported that smoke exposure is well tolerated and resulted in levels of cotinine and carboxyhemoglobin (COHb) similar to what has been reported for smokers [29]. Age-matched control animals were exposed to room air only.

In a second set of experiments, mice (5 per group) were exposed to cigarette smoke for 24 weeks as described above. Subsequently, these mice were exposed to room air only for an additional 60 days (cessation). Age-matched control mice were exposed to room air for the whole duration of the protocol. Survival rate was 100% in all groups.

### Protein Extraction and Western Blots

Gastrocnemius were homogenized in ice-cold lysis buffer with a Polytron® homogenizer. The resulting extract was centrifuged (13 000 rpm, 4°C, 5 min) and the supernatant was transferred to a new tube. An aliquot was reserved for Bradford protein assay and Laemmli buffer was added to the extract. Extracts were boiled for 10 min, aliquoted, and kept at –80°C.

Western blots were performed in duplicate with 10–30 µg total proteins using standard SDS-PAGE procedures. Following transfer onto a nitrocellulose membrane, blotting was completed with the following antibodies from Cell Signaling Technology (Danvers, MA, USA): anti-Akt (#9272, 1∶2000), anti-phospho-Akt (#9271S, 1∶1000), anti-GSK-3β (#9315, 1∶1000), anti-phospho-GSK-3β (#9331, 1∶1000), anti-p70S6K (#9202, 1∶1000), anti-phospho-p70S6K (#9206S, 1∶1000), anti-p38 (#9212, 1∶1000), anti-phospho-p38 (#4511, 1∶1000) and anti-K48-linked polyubiquitin (#4289, 1∶1000). MuRF1 antibody was purchased from GeneTex (#GTX110475, 1∶1000; Irvine, CA, USA). Proteins of interest were detected using a secondary antibody coupled to horseradish peroxidase (#7074, #7076, 1∶5000, Cell Signaling Technology). To ensure equal loading, every result was normalized to tubulin (T5168, 1∶20 000, Sigma-Aldrich, St-Louis, MO, USA).

### Protein Oxidation Assessment

Total protein oxidation was detected using Oxyblot™ Protein Oxydation Detection Kit (Millipore, Billerica, MA, USA) according to the manufacturer’s instructions.

### RNA Extraction and Real-Time PCR

Total RNA was isolated from gastrocnemius using TRIzol® Reagent (Invitrogen, Carlsbad, CA, USA). Reverse transcription was performed using Quantitect™ Reverse Transcription Kit (Qiagen, Valencia, CA, USA) according to the manufacturer’s instructions. Real-Time PCR was performed with the PerfeC_T_a® SYBR® Green SuperMix (Quanta BioSciences, Gaithersburg, MD, USA) in a Rotor-Gene™ 6000 (Qiagen). All qPCR protocols consisted of 1 denaturing cycle at 90°C for 10 minutes, followed by 40 cycles of denaturing at 90°C for 30 seconds, annealing at 60°C for 60 seconds and elongation at 72°C for 60 seconds, followed by final elongation at 72°C for 5 minutes. At the end of the PCR amplifications the samples were subjected to a melting curve analysis. To control for any variations due to efficiencies of reverse transcription and PCR, 18S was used as an internal control. All results were analyzed using the 2^−ΔΔCt^ method [30]. All qPCR runs were performed in triplicate to ensure quantitative accuracy. PCR primer sequences are provided in Table 1.

**Table 1:** Primer sequences used for PCR analyses.

18S	Forward Reverse	TTGACGGAAGGGCACCACCAG GCACCACCACCCACGGAATCG
Atrogin-1	Forward Reverse	TCGGCAAGTCTGTGCTGGTGG CCCAGGCTGACCAGGTCCCG
FoxO3	Forward Reverse	GGTACCAGGCTGAAGGATCA GAGAGCAGATTTGGCAAAGG
IL-1β	Forward Reverse	AGCCTCGTGCTGTCGGACCC TCCAGCTGCAGGGTGGGTGT
IL-6	Forward Reverse	GAGGATACCACTCCCAACAGACC AAGTGCATCATCGTTGTTCATACA
MuRF1	Forward Reverse	CGGCCTGCAGAGGAACCTGC GCACATCGGGTGGCTGCCTT
TNF-α	Forward Reverse	AGCCCACGTCGTAGCAAACCA CATGCCGTTGGCCAGGAGGG
VEGF	Forward Reverse	CCAGGCTGCACCCACGACAG GGCACGCACTCCAGGGCTTC

Definition of abbreviations: FoxO3 = Forkhead box-containing protein O3, IL = Interleukin, MuRF = Muscle RING finger, TNF = Tumor necrosis factor, VEGF = vascular endothelial growth factor.

### Statistical Analyses

**Figure 2:**
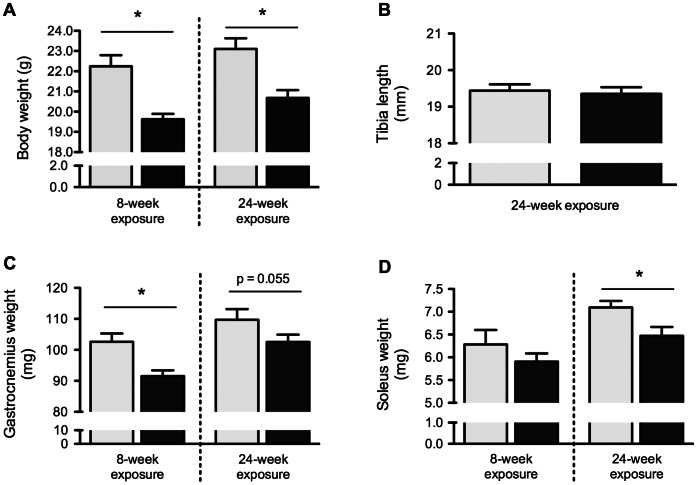
Cigarette smoke exposure decreases whole body weight and skeletal muscle mass. BALB/c mice were either exposed to room air or cigarette smoke twice a day, five days a week, for 8 or 24 weeks. Body weight was measured prior to sacrifice (A). Tibia bones were collected from mice exposed 24 weeks to smoke or room air and measured (B). Gastrocnemius (C) and soleus (D) muscles were excised and weighted. Results are mean ± SEM of 9 or 10 mice. *p<0.05. Room air control mice = Light gray Smoke-exposed mice = Black.

All statistical analyses were performed using Prism 5 (GraphPad Software, La Jolla, CA, USA). Comparisons of experimental conditions were carried out using one-tailed (Figure 2) or two-tailed unpaired t-test. Only mice exposed for the same period of time to experimental conditions were compared in order to avoid any confounding variable induced by aging and whole body growth. P-value <0.05 was considered significant.

## Results

### Impact of Cigarette Smoke Exposure on Muscle Weight and Whole Body Growth

BALB/c mice were exposed to cigarette smoke or room air for 8 or 24 weeks. Whole body mass was reduced by 11.8% (p = 0.007) and 10.5% (p = 0.001) after 8 and 24 weeks, respectively, of exposure to cigarette smoke when compared to mice exposed to room air (Figure 2A). Tibia length was measured as a marker of body growth and no difference was found between room air and smoke-exposed mice after 24 weeks of smoke exposure (Figure 2B) (p = 0.74). Gastrocnemius weight was significantly reduced by 10.8% (p = 0.0018) in mice exposed to cigarette smoke for 8 weeks when compared to room air controls (Figure 2C). Although not statistically significant, a strong tendency toward a 6.5% reduction in gastrocnemius weight was found after 24 weeks of smoke exposure (p = 0.055). Soleus weight was comparable between cigarette smoke and room air exposed mice following 8 weeks of smoke exposure (p = 0.15). Following 24 weeks, soleus weight was 8.7% lower in smoke- compared to room-air exposed mice (Figure 2D) (p = 0.01).

### The Effects of Cigarette Smoke Exposure on Cell Signaling Associated to Protein Degradation

**Figure 3:**
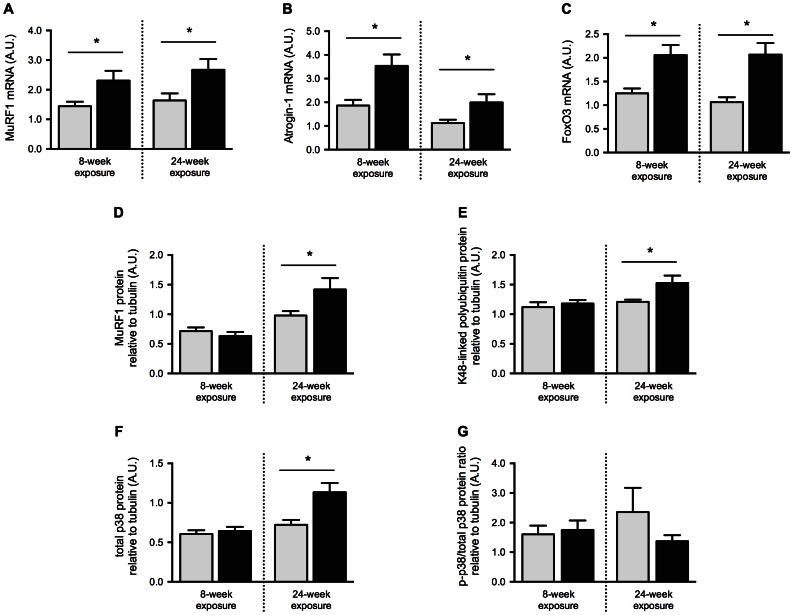
Cigarette smoke exposure time-dependently activates pathways associated to protein degradation in skeletal muscle. BALB/c mice were either exposed to room air or cigarette smoke twice a day, five days a week for 8 or 24 weeks. MuRF1 (A), Atrogin-1 (B) and FoxO3 (C) mRNA expression levels were measured by real-time PCR. Protein levels of MuRF1 (D), polyubiquitin linked to lysine 48 (E), total form of p38 (F) and the ratio of phosphorylated p38/total p38 (G) were assessed by Western Blot and normalized to tubulin signal. All results were obtained with gastrocnemius muscle. Results are mean ± SEM of 9 or 10 mice. *p<0.05 Room air control mice = Light gray Smoke-exposed mice = Black.

Skeletal muscle atrophy-related cell signaling was first evaluated at the mRNA level in gastrocnemius muscles. Expression level of muscle-specific E3-ligases MuRF1 (8 weeks: p = 0.029, 24 weeks: p = 0.0299) and Atrogin-1 (8 weeks: p = 0.0067, 24 weeks: p = 0.034), as well as the transcription factor forkhead box-containing protein O3 (FoxO3) (8 weeks: p = 0.0037, 24 weeks: p = 0.0016), were all increased after both 8- and 24-weeks of cigarette smoke exposure compared to their respective room air controls (Figure 3A, B, C). MuRF1 (p = 0.04), K48-linked polyubiquitin (p = 0.02), and total p38 (p = 0.0051) protein levels were all increased in mice exposed to cigarette smoke for 24 weeks when compared to room air controls (Figure 3D, E, F). No difference was observed following 8 weeks of smoke exposure. Phosphorylated p38/total p38 ratio was not affected by cigarette smoke (8 weeks: p = 0.73, 24 weeks: p = 0.28) (Figure 3G).

### The Effects of Cigarette Smoke Exposures on Cell Signaling Associated to Protein Synthesis

**Figure 4:**
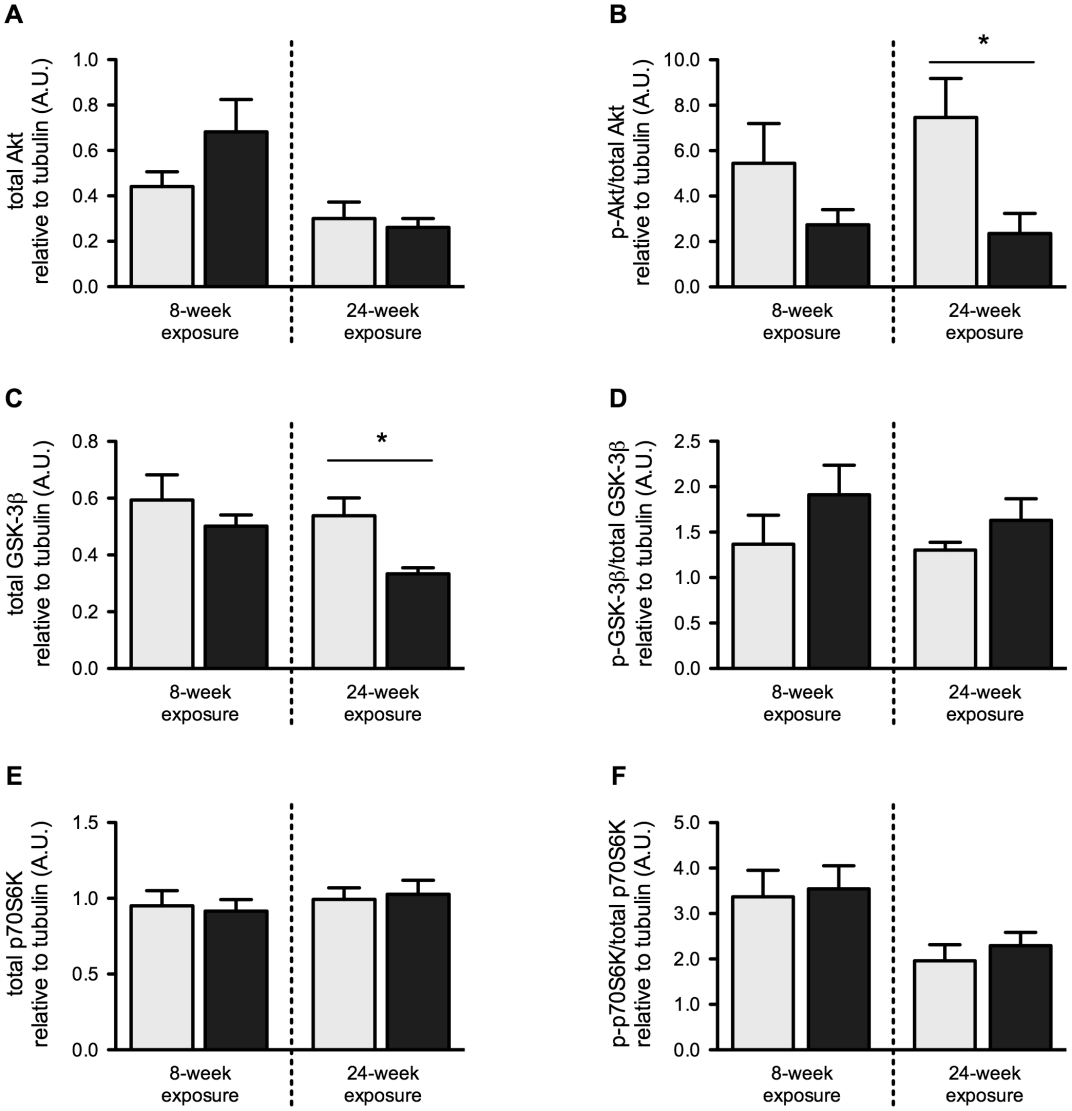
Cigarette smoke exposure time-dependently inhibits pathways associated to protein synthesis in skeletal muscle. BALB/c mice were either exposed to room air or cigarette smoke twice a day, five days a week for 8 or 24 weeks. Protein levels of Akt (A), phosphorylated Akt/total Akt ratio (B), GSK-3β (C), phosphorylated GSK-3β/total GSK-3β ratio (D), p70S6K (E) and phosphorylated p70S6K/total p70S6K ratio (F) were assessed by Western Blot and normalized to tubulin signal. All results were obtained with gastrocnemius muscle. Results are mean ± SEM of 9 or 10 mice. *p<0.05 Room air control mice = Light gray Smoke-exposed mice = Black.

Phosphorylated Akt/total Akt ratio (p = 0.021) and total GSK-3β (p = 0.0087) protein levels were reduced in gastrocnemius muscle collected from mice exposed to cigarette smoke for 24 weeks (Figure 4B, C). Total Akt, phosphorylated-GSK-3β/total GSK-3β, total p70S6K, and phosphorylated-p70S6K/total p70S6K protein ratios were unaffected by both 8 and 24 weeks of smoke exposures (Figure 4A, D, E, F).

### The Effects of Smoking Cessation on Skeletal Muscle Weight and Cell Signaling

**Figure 5:**
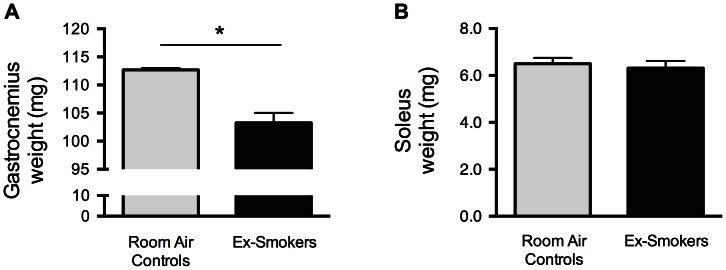
Soleus muscle weight but not gastrocnemius is normalized following smoking cessation. BALB/c mice were either exposed to room air or cigarette smoke twice a day, five days a week, for 24 weeks. All mice were then exposed to room air for an additional 60 days, without smoke exposure. Gastrocnemius (A) and soleus (B) muscles were excised and weighted. Results are mean ± SEM of 5 mice. *p<0.05 Room air control mice = Light gray Smoke-exposed mice = Black.

**Figure 6:**
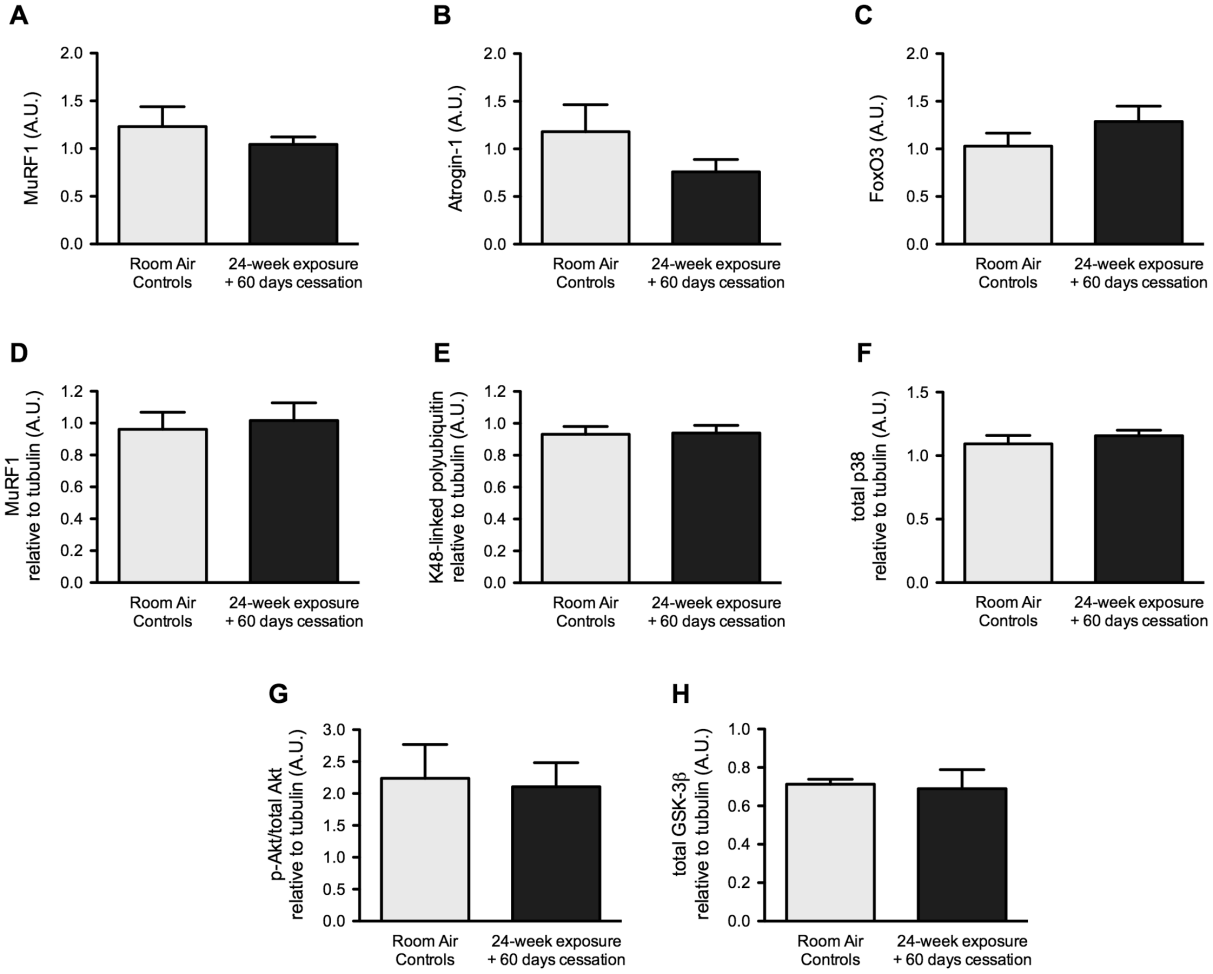
Pro-degradation and anti-synthesis changes induced by chronic cigarette smoke exposure are reversible. BALB/c mice were either exposed to room air or cigarette smoke twice a day, five days a week, for 24 weeks. All mice were then exposed to room air for an additional 60 days, without smoke exposure. MuRF1 (A), Atrogin-1 (B) and FoxO3 (C) mRNA levels were measured by real-time PCR. Protein levels of MuRF1 (D), polyubiquitin linked to lysine 48 (E), total p38 (F), phosphorylated Akt/total Akt ratio (G) and total GSK-3β (H) were assessed by Western Blot and normalized to tubulin signal. Results are mean ± SEM of 5 mice. *p<0.05 Room air control mice = Light gray Smoke-exposed mice = Black.

To document the effects of smoking cessation on gastrocnemius cell signaling, mice were exposed to cigarette smoke for 24 weeks followed by a 60-day smoking cessation period. Gastrocnemius was found to be significantly smaller following smoking cessation compared to room-air controls (p = 0.027), whereas soleus weight was not different between both groups (p = 0.64) (Figure 5A, B). MuRF1 (p = 0.43), Atrogin-1 (p = 0.22), and FoxO3 (p = 0.26) mRNA levels returned to the control level in gastrocnemius after smoking cessation (Figure 6A, B, C). Similarly, MuRF1 (p = 0.73), polyubiquitin (p = 0.91), p38 (p = 0.45), phosphorylated Akt/total Akt ratio (p = 0.85), and total GSK-3β (p = 0.83) exhibited a similar pattern in gastrocnemius following the smoking cessation period (Figure 6D, E, F, G, H).

### The Effects of Smoke Exposures and Smoking Cessation on the Inflammatory, Oxidative and Angiogenic States in Gastrocnemius Muscle

**Figure 7:**
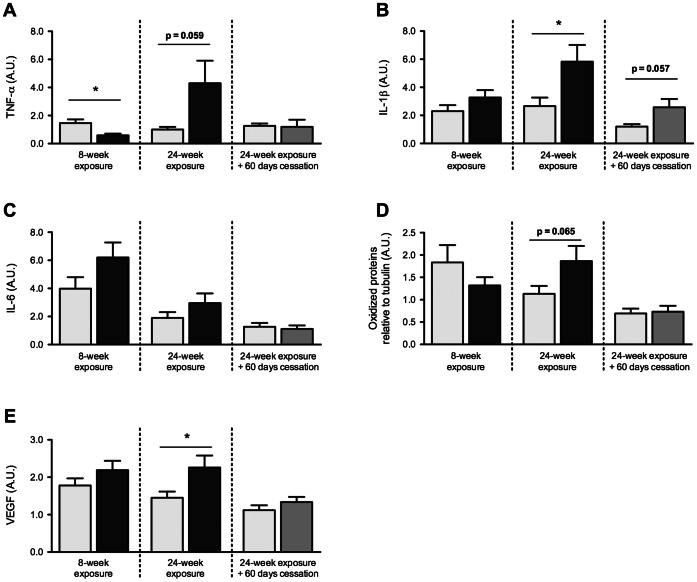
Cigarette smoke exposure induces inflammatory, oxidative and pro-angiogenic states in the skeletal muscle that are partially reversible. BALB/c mice were either exposed to room air or cigarette smoke twice a day, five days a week, for 8 or 24 weeks (9–10 mice per group). Following this protocol, a sub-group (5 mice per group) of control mice and 24-week smoke exposed mice were exposed to room air for an additional 60 days. TNF-α (A), IL-1β (B), IL-6 (C) and VEGF (E) mRNA levels were measured by real-time PCR. Total protein oxidation (D) were assessed by Western Blot and normalized to tubulin signal. Results are mean ± SEM of 5–10 mice. *p<0.05 Room air control mice = Light gray Smoke-exposed mice = Black Ex-smoking mice = Dark gray.

TNF-α mRNA expression level was significantly reduced after 8 weeks of exposure to cigarette smoke (p = 0.0082), while a strong tendency toward the opposite effect was found after a 24-week protocol (p = 0.059) (Figure 7A). Expression of the pro-inflammatory cytokine interleukin-1 beta (IL-1β) was increased in mice exposed to cigarette smoke for 24 weeks (p = 0.03), whereas IL-6 was not significantly different between room air and cigarette smoke-exposed mice (Figure 7B, C). Of the three cytokines measured, only IL-1β mRNA levels strongly tended to remain elevated (p = 0.057) following smoking cessation (Figure 7A, B, C). Total protein oxidation tended to be higher in skeletal muscle of 24-week smoke exposed mice (p = 0.065), a tendency that was abolished by the smoking cessation period (Figure 7D). Finally, VEGF mRNA levels were significantly increased by 24 weeks of cigarette smoke exposure (p = 0.04) and returned to baseline following smoking cessation protocol (Figure 7E).

## Discussion

The objective of this study was to investigate the effects of cigarette smoke exposures on skeletal muscle cell signaling involved in protein synthesis and breakdown, and to assess the reversibility of these effects following smoking cessation. This study emphasizes three key messages. First, the cigarette smoke exposure results in reduced skeletal muscle weight and increases in expression of pro-atrophy related genes. Second, intensity of smoke-induced cell signaling events is increased alongside the total cigarette exposure period. Third, cell signaling returns to control levels when cigarette smoke exposure is stopped for a prolonged period of time.

Mice were exposed to cigarette smoke using a well-characterized whole body smoke exposure system [29]. We previously reported that cigarette smoke exposure is well tolerated and results in cotinine and COHb levels similar to what has been found in human smokers [29]. Cigarette smoke exposure elicits an inflammatory response in the lungs that is characterized by increased mononuclear cells and neutrophils. Mechanistically, this inflammatory response is IL-1α/IL-1R1 dependent [31], but redundant of TNF-α (unpublished data). This model also exhibits similar levels of physical activity, as well as equivalent food and water intake compared to room-air conditions (Debigaré et al, unpublished data).

Despite a decreased body weight after 8 weeks, mice exposed to cigarette smoke exhibited a 5.4% body weight gain between week 8 and week 24. Comparatively, body weight gain was 3.8% in room air-exposed mice over the same period. Mice were not weighed at time zero. Nevertheless, mice were matched for age and gender, and female Balb/c mice of similar age purchased from the supplier (Charles Rivers) typically average 17–18 grams. Therefore, the reported body weight difference is probably the consequence of a failure to gain weight normally during the first weeks of cigarette smoke exposure. The fact that tibia length was similar in smoke-exposed mice and in controls after 24 weeks reveals that the overall skeletal growth was comparable in both conditions. Consequently, the discrepancies in body weight originate from modifications in body composition (i.e. fat versus fat-free mass). Unfortunately, our data do not allow distinction between fat and fat-free mass distribution. Body composition measurements are therefore needed to further document the impact of cigarette smoke exposures on the dynamic and compartmentalized nature of body weight loss in this model.

The effects of cigarette smoke exposures on muscle mass were different relative to the muscle group being assessed. Gastrocnemius, a predominantly glycolytic muscle, was significantly smaller in 8-week smoke exposed mice when compared to their counterparts, whereas only a tendency toward significance was found between both groups after 24 weeks (p = 0.055). Conflicting reports exist concerning the effects of cigarette smoke exposure on gastrocnemius weight [10], [11], [12], [13]. This discrepancy is most probably related to the methodological divergences that exist in the smoke exposure protocols currently used in the literature; e.g. nose-only versus whole body exposure, number of daily exposures, number of days/week, total duration of the protocols, type of cigarettes, animal gender, etc. The fact that gastrocnemius mass was more severely impaired after an 8-week smoke exposure than after 24 weeks (10.8% versus 6.5% decreases) is intriguing. De Paepe reported a type IIa to type IIb fiber switch in gastrocnemius muscle of 18-week smoke-exposed mice [11]. Although speculative, this fiber type transition from hybrid to glycolytic fibers could be the result of an adaptive period where muscle growth is altered, perhaps during the first 8 weeks of the protocol. Following this period, the adapted (more glycolytic) gastrocnemius muscle would then gain mass at a similar rate to non-smoking mice, as seen during the 8 to 24 weeks interval in our study. The soleus, a phasic and predominantly oxidative muscle, was significantly smaller in smoke-exposed mice after 24 weeks. This result confirms previous studies reporting a reduction in soleus weight after 18 weeks [11] and 24 weeks [10] of smoke exposure, although that latter study only reported a strong statistical tendency (p<0.06). It should be noted that nose-only smoke exposures failed to produce soleus muscle weight reduction in 3 and 6 months protocols [12]. With our data, it is impossible to conclude whether the reduction in muscle weight represents atrophy (specific loss of mass and fibers) or failure to gain weight. However, both atrophy and failure to gain weight are likely responses to increased expression of pro-atrophy genes due to smoke exposure. Taken together, our results on muscle weight highlight a differential effect of cigarette smoke exposure on muscle groups, a variance that could be explained by myofibrillar composition or muscle function (postural versus mobility). Specific investigations are required to explore this notion.

When mice were exposed to cigarette smoke for 8 weeks, pro-atrophic genes (i.e. Atrogin-1, MuRF1 and FoxO3) [22], [23] exhibited higher mRNA expression levels when compared to controls, despite the fact that an emphysema-like phenotype and airway remodeling are observed only after prolonged exposure, usually following 4–6 months of cigarette smoke exposure [32]. The effects of cigarette smoke exposure on cell signaling were exacerbated in a 24-week protocol where, in addition to gene expression alterations, intra-muscular protein levels and phosphorylation status of members of the PI3K/Akt and ubiquitin-proteasome pathways were altered. We observed hypophosphorylation of Akt when mice were exposed to cigarette smoke for 24 weeks. Given the central role of Akt in the control of global protein synthesis [24], these results intuitively point toward an altered synthesis process. To clarify this assertion, we analyzed direct and indirect downstream targets of Akt (i.e. GSK-3β and p70S6K respectively) in the same group of mice and found no difference in the phosphorylation ratio of both proteins. However, the total form of GSK-β, an inhibitor of protein translation when unphosphorylated [25], was reduced in smoke-exposed mice. Because the phospho-GSK-3β/total GSK-3β ratio was unaltered in smoke-exposed animals, the net result of the decreased total GSK-3β is a reduction in both phosphorylated and unphosphorylated forms of this protein, suggesting that protein synthesis could be favored in the gastrocnemius (i.e less unphosphorylated GSK-3β). Since Akt and p70S6K phosphorylation status were either decreased or unaltered in smoke exposed mice, we believe that restoration of the protein synthesis process to a level comparable to non-smokers is unlikely with the sole reduction in total GSK-β. Given the fact that protein synthesis rates were not directly measured in this study, these assumptions are speculative and remain to be proven. After 24 weeks of exposure, Akt hypophosphorylation was associated with an altered control of the degradation process, as depicted by increased MuRF1, Atrogin-1, and FoxO3 mRNA levels, as well as MuRF1 and polyubiquitin protein content upregulation. Comparatively, at week 8, increased MuRF1, Atrogin-1 and FoxO3 mRNA levels were observed alongside decreased phospho-Akt/total Akt ratio and increased total Akt (both not significant). As the resulting situation is unclear, we cannot affirm that changes in Akt activity were related to atrophy gene expression at this time-point. Other transcription factors or signaling pathways are likely involved. The fact that gastrocnemius was able to gain weight at a normal rate between week 8 and 24 in smoke-exposed mice remains intriguing, considering that muscle protein degradation signals were higher in these mice at week 24. These results are compatible with a situation where the protein pool present in muscle cells are either more prone to abnormal folding during the synthesis process, are more sensitive to oxidation, or are vulnerable to damage. These protein alterations are known to activate the ubiquitin-proteasome system. Interestingly, our group [33] and others [34] have reported preservation of the phosphorylation status of GSK-3β and p70S6K along with higher expression levels of pro-atrophic markers in the vastus lateralis muscle of COPD patients when compared to matching healthy controls. This situation highlights the relevance of our chronic smoke-exposure mouse model in order to understand the skeletal muscle dysfunction occurring in the context of COPD.

Our results indicate that a pro-inflammatory state in muscle tissue was induced by chronic cigarette smoke exposure, at least at the mRNA level. Systemic and local inflammation is among the most frequently cited underlying mechanism that could contribute to the development of skeletal muscle dysfunction in COPD [35]. In our hands, evidence of increased protein oxidation in smoke-exposed mice was concurrent with an inflammatory state after 24 weeks of exposure, which is in agreement with other reports [15], [16]. In support, it has been shown recently that cigarette smoke exposure in mice elicits changes in cellular redox status in lung tissue [36], as well as accumulation of reactive carbonyls, 4-hydroxy-2-nonenal and malondialdehyde protein adducts in respiratory and limb muscles [16]. Since the buildup in oxidized products was seen late in our model, one could speculate that either exhaustion of the antioxidant defense or proteolytic pathways saturation (e.g. proteasome, autophagy) contributed to this situation. Further studies are needed to investigate the dynamic interactions between production, quenching, and degradation of oxidized products [36].

Perhaps the most striking result of this study is the demonstration of the reversible nature of the smoke-induced skeletal muscle cell signaling perturbations upon smoking cessation. All modifications in regards to protein quantity and phosphorylation status were improved by a smoking cessation period of 60 days. The same phenomenon was observed with mRNA expression levels of all tested genes except IL-1β, a cytokine that remained over-expressed in ex-smoking mice. IL-1β is a pro-inflammatory cytokine known to induce catabolism when incubated with fully differentiated skeletal muscle cells [37]. The result obtained with this cytokine could be a direct consequence of the persistent pulmonary inflammation present after smoking cessation (unpublished data) or an inflammatory process taking place in muscle tissue, independently of TNF-α and IL-6. Nevertheless, these hypotheses were untested in this study and remain to be investigated. Interestingly, gastrocnemius and soleus muscle masses displayed different responses to smoking cessation. Gastrocnemius muscle was found to be 8.3% smaller in ex-smoking mice when compared to room air controls, a value very similar to the result obtained when smoke-exposed mice and room air controls at 8 (-10.8%) and 24 weeks (-6.5%) were compared. Cell signaling was similar in gastrocnemius between groups after smoking cessation. Combination of gastrocnemius muscle weight data and cell signaling results strongly suggests that smoking cessation was associated with a normal muscle protein turnover resulting in normalized muscle homeostasis rather than a muscle mass expansion that would translate into a similar muscle mass between controls and ex-smokers. Surprisingly, soleus muscle was able to normalize its weight after the cessation period. Intuitively, one would not expect 10 months old mice to display muscle growth. The reason why soleus was able to gain weight in the smoke-free period may reside in the metabolic profile of this muscle. Despite the fact that soleus exhibits a fiber shift from type IIa to type IIb in smoke-exposed mice [10], [12], [13], this muscle is still largely composed of oxidative fibers under smoking conditions. This oxidative-dominant phenotype perhaps makes this muscle prone to gain weight when the systemic environment normalizes under smoking cessation conditions. Since data on soleus cell signaling is lacking, we cannot speculate whether soleus cell signaling was in an anabolic or catabolic state during the 60-day smoking cessation period. Further study of the soleus is needed to understand the exact mechanisms.

In conclusion, cigarette smoke perturbs cellular signaling in limb muscle tissue in as few as after 8 weeks of smoke exposure. Disruption in cellular signaling persist and broaden as the exposure to cigarette smoke is prolonged, suggesting further alterations in muscle function. Interestingly, almost every muscle signaling modifications examined in this study were reversible following smoking cessation, opening a door for further studies aimed at understanding the relationship between inhaled cigarette smoke and systemic stresses.
